# Development and validation of a scale for streaming dependence (SDS) of online games in a Peruvian population

**DOI:** 10.3389/fpsyg.2023.1184647

**Published:** 2023-08-24

**Authors:** Liset Z. Sairitupa-Sanchez, Alejandra Collantes-Vargas, Oriana Rivera-Lozada, Wilter C. Morales-García

**Affiliations:** ^1^Escuela Profesional de Psicología, Facultad de Ciencias de la Salud, Universidad Peruana Unión, Lima, Peru; ^2^South American Center for Education and Research in Public Health, Universidad Norbert Wiener, Lima, Peru; ^3^Escuela de Medicina Humana, Facultad de Ciencias de la Salud, Universidad Peruana Unión, Lima, Peru; ^4^Escuela de Posgrado, Universidad Peruana Unión, Lima, Peru; ^5^Facultad de Teología, Universidad Peruana Unión, Lima, Peru

**Keywords:** streaming, dependence, validation, games, online

## Abstract

**Background:**

Addiction to online video game streaming has become one of the most appealing ways to occupy leisure time and is one of the most popular activities. The satisfaction it provides and the time invested in it are two of the main reasons why it is preferred. However, despite the clear benefits that this activity offers, in some cases, excessive use can lead to personal and/or family problems or abuse.

**Objective:**

The objective of the study was to develop and validate a scale to measure potential traits of dependence on online game streaming. The participants were 423 Peruvian adults aged between 18 and 47 years (*M* = 22.87, SD = 5.02). The Streaming Dependence Scale (SDS) was developed based on a literature review, and exploratory factor analysis (EFA) and confirmatory factor analysis (CFA) were conducted.

**Results:**

The scale showed adequate internal consistency (α, CR, ω, and H > 80). Confirmatory analysis confirmed the one-dimensional structure (χ^2^ = 10.250, df = 5; *p* = 0.068; CFI = 0.98, TLI = 0.96, RMSEA = 0.06, SRMR = 0.05).

**Conclusion:**

The brief SDS is a valid and reliable measure that can be used as a useful tool to identify and evaluate streaming dependence.

## 1. Introduction

In the age of digital transformation, traditional leisure patterns have undergone a profound metamorphosis. Video games and streaming platforms have become central players in this transformed landscape, altering youth interaction and socialization (Hu et al., 2017; Appel et al., 2020; Li et al., 2022; Islam et al., 2023). This technological revolution has prompted a deep rethinking of the concepts of leisure and entertainment, and their implications for personal growth and socialization (Jackson et al., 2011; Kaimara et al., 2022).

However, video game addiction is an emerging phenomenon that has become increasingly worrisome, given its effects extend to individual aspects disrupting family and social dynamics (Choìliz and Marco, 2016). Video games, once seen as an experimental form of entertainment, have evolved into an integral part of many people’s daily lives, especially among the youth (Boris, 2019; Dwivedi et al., 2022). While video games can be useful tools for cognitive and coordinative development, their excessive use can be detrimental, manifesting in addictions, social isolation, promotion of anger and violence, and harmful effects on physical and mental health (Zamani et al., 2009; Agbaria, 2022; T’ng et al., 2022; Zaman et al., 2022; Lérida-Ayala et al., 2023). Video game addiction is an integral part of Internet Gaming Disorder (IGD) and is emerging as a relevant challenge in the field of mental health (VandenBos and American Psychological Association, 2015). It manifests as a recurrent and poorly adapted pattern of gaming behaviors, continuing despite evident negative consequences. This addiction roots in motivational control issues and shares characteristics with gambling addiction (Kuss, 2013). The Diagnostic and Statistical Manual of Mental Disorders (DSM-5) defines video game addiction as a constant and compulsive use of the internet for gaming, leading to clinically significant distress and psychological changes (Morrison, 2015). Studies show this addiction can induce alterations in specific brain areas like the prefrontal cortex, the ventral striatum, and the dorsal striatum (Kuss and Griffiths, 2012). These neurophysiological changes can manifest in a variety of symptoms ranging from sleep disorders and fatigue to anxiety and depression, affecting both the individual’s mental and physical health (Kircaburun et al., 2018).

Video game addiction has given rise to an even more worrying phenomenon: the addiction to video game streaming. The popularization of platforms like Twitch, which hosts an average daily audience of 26.5 million viewers, has shown a concerning increase in addiction to these streaming services (Speed et al., 2023). In this regard, addiction to online video games and, more recently, to their live streaming, have exacerbated the problem, increasing the risk of developing dependency (Diotaiuti et al., 2022b). The DSM-IV (1995) defines dependency as a pattern of video game use that causes significant impairment or distress. This manifests in various aspects, including an increase in tolerance, presence of withdrawal symptoms, excessive dedication to gaming, an uncontrollable craving to play, neglect of other vital activities, and difficulties limiting their use. In the DSM-5, Internet Gaming Disorder (IGD) has been introduced as a related entity that needs to be studied in greater depth (Morrison, 2015). Choìliz and Marco (2016) expand this definition by describing dependency as the excessive and inappropriate use of an initially enjoyable activity but that eventually generates serious personal and family complications. Thus, addiction to video game streaming can have severe repercussions for daily life, emotional stability and has been associated with impulsivity and codependency (Diotaiuti et al., 2022b). Susceptibility to streaming addiction is particularly acute among the youth. Young people who are entangled in the web may experience distress when denied access, which can trigger mental health problems like depression and rumination (Diotaiuti et al., 2022a). The proliferation of online video game streaming amplifies this dependency, especially when intertwined with real-time streams, increasing the risk of developing dependency (Li et al., 2020). Therefore, addiction to streaming or the internet can disrupt daily life management, relationships, and emotional stability. This dependency, often concurrent with other psychiatric symptoms and disorders, is associated with impulsivity and codependency. The similarities between video game addiction and addictive and obsessive-compulsive disorders highlight the need to consider different subtypes of addiction for adequate treatment (Petruccelli et al., 2014).

Video game and streaming addiction is not a uniform experience and can vary enormously from one place to another. An example of this is found in Peru, where the use of the internet and video games has risen in recent years, particularly among the youth (INEI, 2023). Despite this growth, there is a significant gap in specific research on video game and streaming addiction in Peru, highlighting the need for a more detailed examination to fully understand this phenomenon in specific cultural and socioeconomic contexts. The analysis of streaming services and the interactive dynamics between streamers and the audience becomes a critical lens to unravel and combat the dependency on online video game streaming. Both, streamers and viewers, may experience a loss of control and seek to fulfill their affective needs through live streams, thereby fostering dependency (Sussman and Sussman, 2011; Choìliz and Marco, 2016). These factors can be influenced by a multitude of elements, among them, the way the streamer interacts with their audience, which can further reinforce this addictive bond (Sjöblom and Hamari, 2017; Zhao et al., 2018).

However, it is essential to note that although video game and streaming addiction present significant risks, it does not entirely discredit the potential benefits these services can offer. Streaming services can provide significant benefits in terms of learning, skill development, and social support (Li et al., 2020). Nevertheless, addressing the excessive and inappropriate use of these services is crucial, as it can stoke internet addiction and streaming dependency, leading to severe personal and family implications (Choìliz and Marco, 2016; Diotaiuti et al., 2022b). This streaming dependency can be linked to other psychiatric disorders, underlining the need to consider a variety of factors when addressing this addiction (Diotaiuti et al., 2022a). In Peru, as in many other countries, there are no national data shedding light on the prevalence of video game streaming addiction. However, it is reasonable to postulate that this problem is on the rise, considering the increasing popularity of video games and video game streaming platforms in these geographies. It is imperative that more research is conducted to decipher the risk factors and consequences of video game and video game streaming addiction, to conceive effective prevention and treatment strategies. This task is of critical importance in a world where video games and video game streaming are becoming an increasingly integrated part of the daily lives of countless people worldwide.

Therefore, the aim of this study is to develop and evaluate the psychometric properties of a scale for live-streamed online gaming addiction in adults.

## 2. Materials and methods

### 2.1. Design and participants

The present study, of an instrumental nature (Ato et al., 2013), was based on convenience sampling. For sample selection, we relied on an electronic calculator (Soper, 2022), considering variables such as: the number of observed and latent variables in the model, the anticipated effect size (λ = 0.10), the desired statistical significance (α = 0.05), and the level of statistical power (1–β = 0.90). With these criteria, the minimum required sample was 199 participants. However, we expanded the sampling and managed to recruit a total of 423 Peruvian adults from the three regions of the country (coast, highlands, jungle). The participants’ ages ranged between 18 and 47 years (*M* = 22.87, SD = 5.02). The study’s inclusion criteria stipulated that participant had to be over 18 years old, living in Peru, and have regular access to video game streaming platforms. We excluded individuals who did not meet the age and residency criteria, as well as those who did not have regular access to streaming platforms. The majority of participants (Table 1) in our sample were men (61.5%). In terms of geographical distribution, most lived in the coastal region of the country (71.4%). In terms of living arrangements, those living with their parents predominated (38.1%). As for the level of education, it was found that the largest proportion of respondents had an incomplete higher education (40.7%).

**TABLE 1:** Demographic characteristics.

Characteristics		*n*	%	% Accumulated
Gender	Female	163	38.5	38.5
	Male	260	61.5	100
Region	Coast	302	71.4	71.4
	Mountain	97	22.9	94.3
	Jungle	24	5.7	100
Living with	Alone	92	21.7	21.7
	Parents	161	38.1	59.8
	Partner	31	7.3	67.1
	Other relatives	71	16.8	83.9
	Friends	68	16.1	100
Education	Incomplete secondary	38	9	9
	Completed secondary	94	22.2	31.2
	Incomplete higher	172	40.7	71.9
	Completed higher	119	28.1	100

### 2.2. Instruments

#### 2.2.1. Streaming dependence

The meticulously designed Streaming Dependence Scale (SDS) was created based on a rigorous literature analysis. This analysis took into account the established diagnostic criteria present in the Diagnostic and Statistical Manual of Mental Disorders, fourth (DSM-IV, 1995), and fifth editions (DSM-V), as well as integrating findings from previous studies focused on gaming addiction (Choìliz and Marco, 2016). The literature exploration confirmed a preliminary set of 10 evaluative items. This initial scale underwent expert evaluation by three clinical psychologists who made adjustments based on their deep knowledge and experience in the field. These items assess multifaceted aspects of streaming dependence, covering the following domains: (a) salience, which refers to the amount of time individuals dedicate to streaming consumption and the extra time invested in related activities; (b) mood modification, considering whether the individual feels they are spending too much time watching streaming; (c) tolerance, evaluating if they can watch streaming for more than 3 h straight without being bothered by the lack of attention to other people or responsibilities; (d) withdrawal symptoms, analyzing if the individual primarily uses their free time for streaming consumption; (e) conflict, whether there have been arguments with loved ones due to the time spent in front of the screen; and (f) relapse, understood as sleep loss or decreased hours of rest as a result of streaming consumption. The items were crafted with three response options, following a Likert scale format, where “Almost never” corresponds to 1, “Sometimes” to 2, and “Almost always” to 3. This way, an assessment is achieved that reflects the individual nuances of streaming dependence, emphasizing personalization and detail in its study. The SDS, as an evaluation tool, aims to provide a quantifiable and reproducible measure of streaming dependence from an individual perspective. Its use allows for a deeper understanding of this emerging phenomenon, opening new avenues for research and clinical intervention (DSM-IV, 1995; Choìliz and Marco, 2016; DSM-V). By applying it, the goal is to gain a more profound understanding of how the digital world, and more specifically streaming, affects people’s daily lives and mental health.

#### 2.2.2. Generalized anxiety

To assess anxiety, the Spanish version of the Generalized Anxiety Disorder Scale, GAD-2 (García-Campayo et al., 2012), was employed. This scale is a concise and abbreviated adaptation of the more extensive GAD-7, specifically designed to provide a quick and efficient measurement tool. The GAD-2 consists of two items, and its response scale follows a Likert-type format, with options ranging from 0 = never to 3 = almost every day. The version adapted to Spanish in Peru was used, with a reliability for internal consistency of α = 0.738 (95% CI, 0.699, 0.773) (Merino et al., 2017).

### 2.3. Procedure

This study faithfully adhered to the principles of integrity, transparency, and respect for human dignity, ensuring the fidelity and accuracy of the findings. The Ethics Committee of a Peruvian university (reference 2181-2022/UPEU-FCS-CF) meticulously examined and approved our research protocol. Each participant was fully and clearly informed about the nature of our study. Informed consent met legal requirements and reflected our conviction in respecting the individual’s autonomy and the right to decide about their participation in the research. Data collection was conducted in person, underscoring to each participant that their involvement in the study was entirely voluntary and anonymous.

### 2.4. Data analysis

We began our analysis with a total sample of participants divided into two groups to facilitate robust cross-validation. Sample 1, with 162 participants, served as the basis for developing the model in the exploratory factor analysis (EFA), while Sample 2, made up of 261 participants, was used to validate the model through confirmatory factor analysis (CFA) (VandenBos and American Psychological Association, 2015). A descriptive analysis of the items of the Streaming Dependency Scale (SDS) was carried out, calculating the mean, standard deviation, skewness, kurtosis, and performing a corrected inter-test correlation analysis. Skewness (g1) and kurtosis (g2) metrics were considered appropriate when values ranged between ± 1.5 (Pérez and Medrano, 2010). We implemented the corrected item-test correlation analysis to remove items when r(i-tc) < = 0.2 or when multicollinearity with (i-tc) < = 0.2 was detected (Kline, 2016).

To examine the factorial structure of the SDS, an EFA was performed using unweighted least squares with oblique rotation (promax). Parallel analysis allowed us to determine the optimal number of factors. We verified the suitability of the data with Bartlett’s test of sphericity and the Kaiser-Meyer-Olkin (KMO) coefficient (Kaiser, 2016; Worthington and Whittaker, 2016).

Once the number of factors in the EFA was established, we proceeded with the CFA on the unifactorial scale using the WLSMV estimator, known for its robustness against deviations from inferential normality (Muthen and Muthen, 2017). We evaluated the model fit using the chi-square test (χ^2^), the Confirmatory Fit Index and Tucker-Lewis index (CFI and TLI ≥ 0.95) (Schumacker and Lomax, 2016), and the Root Mean Square Error of Approximation and Standardized Root Mean Square Residuals (RMSEA and SRMSR ≤ 0.05) (Kline, 2016). Additionally, to demonstrate internal validity through convergent validity, we calculated the average variance extracted (AVE) per factor (AVE > 0.50), which indicates that more than 50% of the variance is due to its indicators (Fornell and Larcker, 1981). We evaluated external validity by measuring the latent relationship between streaming dependence and anxiety through structural equation modeling (SEM).

Finally, we evaluated internal consistency using Cronbach’s alpha coefficient, composite reliability (CR), McDonald’s omega coefficient (McDonald, 1999), and the H coefficient (Hancock and Mueller, 2001), looking for values above 0.70 (Hancock and Mueller, 2001).

All statistical analysis was performed using R software 4.1.1 (R Foundation for Statistical Computing, Vienna, Austria).^1^

## 3. Results

### 3.1. Content validity

Table 2 shows the results of the evaluation of the experts who analyzed the relevance, coherence, clarity, and context of the items of the SDS scale. It can be seen that items 1, 2, 3, 4, 6, 8, 9, and 10 received a favorable evaluation with a score greater than 0.80. However, items 5 and 7 received a score lower than 0.80, so they were decided to be eliminated based on the criteria established (Escurra Mayaute, 1988).

**TABLE 2:** Aiken V for the evaluation of the SDS items.

Items	Relevance	Coherence	Clarity	Context
1	1.00.00	1.00	1.00	1.00
2	1.00	1.00	1.00	1.00
3	1.00	1.00	1.00	1.00
4	1.00	1.00	1.00	1.00
5	1.00	1.00	0.60	0.67
6	1.00	1.00	1.00	1.00
7	1.00	0.70	0.78	0.89
8	1.00	0.83	1.00	1.00
9	1.00	1.00	0.89	1.00
10	1.00	1.00	1.00	1.00

### 3.2. Descriptive statistics of the items

In Table 3, the results of the descriptive statistics of the scale items are presented. It can be seen that item 6 had the highest mean (*M* = 1.65), while item 2 had the lowest mean (*M* = 1.44). In terms of dispersion, it was found that item 6 (SD = 0.65) had a higher dispersion compared to the other items. Skewness (g1) and kurtosis (g2) fluctuated within the acceptable values of ± 1.5 for all items, suggesting a multivariate normal distribution. In addition, it was found that the scale had item-total correlations greater than 0.30, indicating high homogeneity. Finally, an acceptable internal consistency was obtained for each item by calculating the Cronbach’s alpha, with values greater than 0.70 (>0.70).

**TABLE 3:** Descriptive statistics and reliability.

Items	*M*	sd	g1	g2	r.cor	α
Actualmente dedico más tiempo a ver streaming que cuando comencé.	1.55	0.63	0.7	−0.53	0.52	0.76
Siento que estoy dedicando demasiado tiempo a ver streaming.	1.44	0.59	0.94	−0.14	0.52	0.76
He llegado a ver streaming por más de tres horas consecutivas sin preocuparme por otras responsabilidades o personas (familia o amigos).	1.46	0.6	0.91	−0.2	0.53	0.76
He tenido discusiones con familiares o amigos debido a mi tiempo dedicado a ver streaming.	1.46	0.57	0.77	−0.44	0.45	0.77
He perdido el sueño o he dormido menos debido a ver streaming.	1.48	0.62	0.91	−0.22	0.51	0.76
Utilizo mi tiempo libre para ver streaming.	1.65	0.65	0.5	−0.73	0.48	0.77
He mentido a alguien que quiero acerca de la cantidad de tiempo que dedico a ver streaming.	1.46	0.63	1.02	−0.07	0.52	0.76
Dedico tiempo extra a temas relacionados con el streaming, incluso cuando no lo estoy viendo (busco información, hablo con amigos y familiares, etc.).	1.63	0.68	0.6	−0.74	0.43	0.78

g1, skewness; g2, kurtosis; r.cor, item-total correlations; α, Cronbach’s alpha.

### 3.3. Internal structure evidence

**FIGURE 1:**
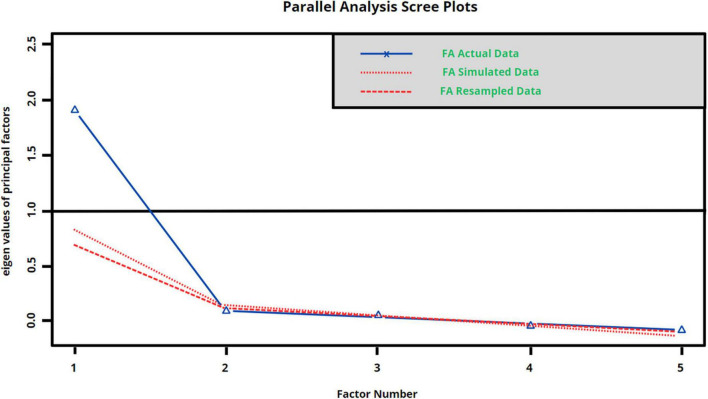
Parallel analysis.

The exploratory factor analysis (EFA) was carried out on the unidimensional scale to determine the underlying structure of the items. The results indicated an adequate fit of the data through the KMO coefficient (0.83) and the Bartlett’s test of sphericity (*p* < 0.001). Parallel analysis and the scree plot suggested the extraction of a single factor (Figure 1). The maximum likelihood extraction method and the varimax rotation method eliminated iteratively the items whose loads were lower than 0.50 in the proposed factors and with individual communality with loads lower than required (h2 < 0.30) (Costello and Osborne, 2005; Lloret-Segura et al., 2014) therefore it was considered to eliminate item 4, 6, and 8 (Table 4).

**TABLE 4:** EFA, CFA, AVE, and reliability.

			AFC	
AFE			Model 1	Model 2
Items	F1	h2	F1 (λ)	F1 (λ)
S1	0.62	0.38	0.71	0.74
S2	0.61	0.38	0.75	0.77
S3	0.62	0.38	0.66	0.68
S4	0.49	0.24	0.72	
S5	0.56	0.31	0.65	0.59
S6	0.53	0.28	0.39	
S7	0.62	0.38	0.64	0.61
S8	0.47	0.22	0.43	
% variance	22.6			
α			0.83	0.81
CR			0.83	0.80
ω			0.84	0.83
H			0.86	0.80
AVE			0.40	0.56
χ^2^			65.81	10.25
gl			20	5
CFI			0.91	0.98
TLI			0.87	0.96
RMSEA			0.09	0.06
SRMR			0.05	0.03

α, Cronbach’s Alpha; ω, McDonald’s omega; H, H coefficient; λ, factor load; AVE, average variance extracted.

### 3.4. Validity based on internal structure

Insights derived from the exploratory factor analysis (EFA) allowed for the development of the confirmatory factor analysis (CFA) to assess the factor structure (Table 2). Initially, a model incorporating all items was performed. However, the goodness-of-fit indices [χ^2^ = 65.81, df = 20; *p* = 0.000; CFI = 0.91, TLI = 0.87, RMSEA = 0.09 (90% CI 0.07–0.065), SRMR = 0.05] did not reach the standards, indicating that this model might not be the optimal representation of our data structure. In addition, the factor loadings (λ) of items 6 and 8 were below the threshold (>0.50), suggesting that these items might not be strongly associated with the underlying factor. In light of these observations, a second model was developed, taking into account the preliminary evidence provided by the EFA. This analytical step indicated that the goodness-of-fit indices were adequate [χ^2^ = 10.250, df = 5; *p* = 0.068; CFI = 0.98, TLI = 0.96, RMSEA = 0.06 (90% CI 0.07–0.065), SRMR = 0.03], thus providing a more robust argument for the validity of our model. Furthermore, factor loadings exhibited satisfactory magnitudes (λ > 0.50), reinforcing our confidence in the role of each item in representing the underlying construct (Appendix).

### 3.5. Validity with convergent and reliability

In relation to internal consistency (Table 4) in Model 1, the alpha (α), omega (ω), and composite reliability (CR) were all above the recommended threshold of 0.70, indicating good internal consistency of the items. The H coefficient was 0.86, also indicating good reliability. However, the average variance extracted (AVE) was 0.40, which is below the recommended threshold of 0.50, suggesting that the items might not converge well on the latent factor. While in Model 2, the reliability indicators (α, ω, CR, and H) were all above 0.70, showing good internal consistency. In addition, the AVE was 0.56, exceeding the recommended threshold of 0.50, suggesting better convergence on the latent factor compared to Model 1.

### 3.6. Relationship between streaming dependence (SDS) and anxiety

**FIGURE 2:**
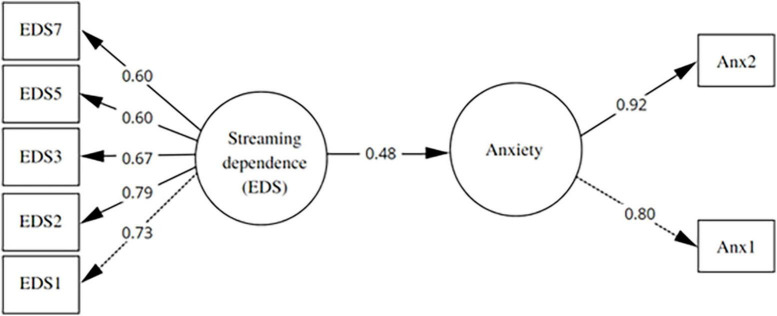
Predictive model of streaming dependence on anxiety.

To evaluate the relationship between the AFC and other constructs, a SEM model was proposed with two latent variables: streaming dependence and anxiety. This model showed a good fit: χ^2^ = 24.16, df = 13; *p* = 0.03; CFI = 0.98, TLI = 0.96, RMSEA = 0.06 (90% CI: 0.02–0.09), SRMR = 0.04 (Figure 2). Additionally, streaming dependence was positively related to anxiety (0.48; *p* < 0.001).

## 4. Discussion

The digital world presents a universe of possibilities and opportunities, but also a vast field of challenges and threats to our physical and mental wellbeing. The use of online games, particularly those streamed live, has witnessed an unprecedented increase, raising concerns related to fatigue, insomnia, anxiety, and depression. The giants of this digital era, such as Twitch and YouTube, have experienced significant growth in their traffic, becoming the primary means of consuming online gaming content. This growing dependence on live-streamed online games on streaming platforms has become an enigma that oscillates between fascination with virtual immersion and fear of digital addiction. However, psychometric evidence supporting the existence of dependence on live-streamed online games, especially in regions like Peru and Latin America, remains scarce and insufficient. In the face of this challenge, the present study aimed to develop and evaluate a new psychometric assessment tool, the Streaming Dependence Scale (SDS).

The SDS was designed to capture the nuances of dependence on live-streamed online games in adults. Drawing inspiration from the video game dependence criteria of the DSM-IV (1995) and DSM-V (Morrison, 2015), as well as previous studies on gaming addiction (Choìliz and Marco, 2016), 10 items were created focusing on key aspects such as salience, mood modification, tolerance, withdrawal symptoms, conflicts, and relapse.

The findings of the Exploratory Factor Analysis (EFA) supported a one-dimensional structure, explaining 22.6% of the variance of the construct. However, the low communalities (h2 < 0.30) of items 4, 6, and 8 suggested their removal to improve the model’s robustness (Costello and Osborne, 2005; Lloret-Segura et al., 2014).

The Confirmatory Factor Analysis (CFA) supported the proposed one-dimensional structure, highlighting the model’s robustness. Although the first model did not show satisfactory fit indices (CFI = 0.91 and TLI = 0.87, RMSEA = 0.09) (Kline, 2016; Schumacker and Lomax, 2016), the second model–after the removal of the mentioned items–demonstrated excellent goodness-of-fit indices (Hu and Bentler, 1999). This implies that the SDS has a robust and suitable factorial structure for future applications.

The Streaming Dependence Scale (SDS) demonstrated high internal consistency, with McDonald’s Omega (ω) and coefficient H values exceeding 0.70 (Raykov and Hancock, 2005; Dominguez-Lara, 2016). This indicates the scale’s reliability in assessing latent variables, solidifying it as a reliable tool for measuring streaming dependence.

Convergent validity of the Streaming Dependence Scale (SDS) was also evaluated through the positive correlation between its items and other indicators of the same variable. Average Variance Extracted (AVE) was used as a measure to determine whether the factors explain a significant portion of the indicators’ variance, with an acceptable threshold of AVE greater than 0.5 (Fornell and Bookstein, 1982; Chin, 1998). In the first evaluated model, AVE was found to be less than 0.50, but in the second model, AVE surpassed the threshold with a value greater than 0.50. Additionally, the convergent validity of the second model was tested with another construct, finding that streaming dependence positively predicts anxiety. This suggests that higher levels of streaming dependence are associated with higher levels of anxiety, similar to previous studies (Lee et al., 2016). However, it is important to note that anxiety was measured in this study with only 2 items assessing the frequency of anxiety, so future studies should consider the duration and severity of anxiety.

### 4.1. Implications

The findings from our study on the Streaming Dependency Scale (SDS) have implications in several areas. First, from a professional practice perspective, the SDS emerges as an essential tool for mental health professionals. Specifically, when dealing with youth and young adults, the SDS offers an effective means to identify and assess dependency levels, paving the way for timely and effective interventions. Therefore, therapists, psychologists, and other healthcare professionals can use the SDS as a primary assessment tool, enabling them to design appropriate interventions and provide better guidance to their patients. Second, given the rising use of platforms such as Twitch and YouTube, streaming dependency is becoming an emerging public health issue. It is imperative that regulators and lawmakers collaborate with these platforms to formulate preventive policies. These policies can include awareness campaigns, limitations on consecutive streaming hours, and education about the risks associated with excessive use. Third, while theories on digital addiction have previously focused on video games and the Internet, the SDS introduces an additional dimension addressing streaming. This gives us a foundation to theorize about the unique characteristics of streaming, its distinct appeal, and how these factors can contribute to the development of a dependency. Fourth, in the realm of educational practice, the SDS could serve as a valuable resource for educators and school counselors. With the increasing integration of digital resources in the classroom, understanding the potential dependency on streaming can inform instructional design. For instance, educators might be cautious when incorporating streaming platforms as part of their curriculum, being aware of the potential for increased dependency.

Moreover, it is crucial to research the applicability of the SDS across different cultural and geographical contexts to ensure its universality. Additional factors, such as real-time social interactions and the relationship between personality, academic performance, social relations, and mental health, should be considered.

### 4.2. Limitations

This study also presents certain limitations that need to be acknowledged. One primary limitation lies in the sample size, especially when advanced analytical techniques are incorporated, which heavily depends on the magnitude of the sample. Various guidelines, such as those proposed by MacCallum et al. (2001), suggest that sample sizes for factor analysis should be considerably large, especially when the number of items is substantial. While Comrey and Lee (2013) have posited that a subject-item ratio of 10:1 might be suitable, some experts argue in favor of even higher ratios to ensure robust and reliable outcomes. For future research, we strongly recommend increasing the sample size. Another limitation is the study’s focus on a single geographical region, potentially constraining the generalizability of the findings to other populations and cultures. Cultural and socioeconomic diversity can significantly influence perceptions and experiences related to streaming dependence. Therefore, it’s recommended that future studies adopt a multicentric design, incorporating participants from a variety of regions. Moreover, even though the study drew inspiration from established diagnostic criteria such as DSM-IV and DSM-V (American Psychiatric Association, 1994, American Psychiatric Association, DSM-5 Task Force, 2013), the ever-evolving nature of digital media consumption implies that continuous adaptation and revision of these criteria are crucial (Griffiths, 2008). Additionally, the reliance on convenience sampling may have limited the generalizability of the results to the entire adult Peruvian population that engages in live-streamed online gaming. Future research should consider utilizing random sampling to procure a sample more representative of the total adult Peruvian population involved in live online game streaming.

## 5. Conclusion

The Streaming Dependence Scale (SDS) offers a valid and reliable tool within the current landscape of digital mental health research. Not only does the SDS allow for a rigorous evaluation of streaming dependence, but it also lays the groundwork for a deeper understanding of how the intrinsic features of live streaming, such as its real-time interactivity and the formation of virtual communities, can influence the onset of addictive behaviors. This understanding, in turn, is crucial for drawing parallels and distinctions between streaming dependence and other recognized forms of digital addiction, like video game addiction or social media addiction. Consequently, it’s imperative to continue research in this direction, not just to assess the prevalence and severity of streaming dependence across various populations and cultural contexts but also to identify associated risk and protective factors. Furthermore, it’s vital to develop, implement, and evaluate evidence-based intervention programs that address both the prevention and treatment of this emerging form of dependence.

## Data availability statement

The raw data supporting the conclusions of this article will be made available by the authors, without undue reservation.

## Ethics statement

The study was carried out by the Ethics Committee of the Universidad Peruana Unión (reference 2181-2022/UPEU-FCS-CF). The studies were conducted in accordance with the local legislation and institutional requirements. The participants provided their written informed consent to participate in this study. Written informed consent was obtained from the individual(s) for the publication of any potentially identifiable images or data included in this article.

## Author contributions

LS-S, AC-V, and WM-G participated in the conceptualization of the idea. LS-S and WM-G were in charge of the methodology and software and commissioned the data curation, and resources. All authors contributed the validation, formal analysis, and research, carried out the writing of the first draft, review and editing, visualization, and supervision and have read and approved the final version of the manuscript.

## Footnotes

1
http://www.R-project.org

## Appendix

**APPENDIX TABLE 1:** Streaming Dependence Scale (EDS) in Spanish.

Ítem	Casi nunca	A veces	Casi siempre
1. Actualmente dedico más tiempo a ver streaming que cuando comencé.	1	2	3
2. Siento que estoy dedicando demasiado tiempo a ver streaming.	1	2	3
3. He llegado a ver streaming por más de tres horas consecutivas sin preocuparme por otras responsabilidades.	1	2	3
4. He perdido el sueño o he dormido menos debido a ver streaming.	1	2	3
5. He mentido a alguien que quiero acerca de la cantidad de tiempo que dedico a ver streaming.	1	2	3

Escala de Dependencia al Streaming (EDS) en español. A continuación, encontrarás una serie de afirmaciones relacionadas con tu comportamiento en torno al uso de plataformas de streaming. Lee atentamente cada una y evalúa la frecuencia con la que te ocurre cada situación. Para cada ítem, debes seleccionar una de las tres opciones que mejor se ajuste a tu experiencia.
